# In Vitro Screening of a 1280 FDA-Approved Drugs Library against Multidrug-Resistant and Extensively Drug-Resistant Bacteria

**DOI:** 10.3390/antibiotics11030291

**Published:** 2022-02-22

**Authors:** Lucie Peyclit, Sophie Alexandra Baron, Linda Hadjadj, Jean-Marc Rolain

**Affiliations:** 1 Aix Marseille University, IRD, APHM, MEPHI, 19-21 Boulevard Jean Moulin, CEDEX 05, 13385 Marseille, France; peyclit.lu@gmail.com (L.P.); sophiebaron363@gmail.com (S.A.B.); linda.hadjadj@univ-amu.fr (L.H.); 2 IHU-Méditerranée Infection, 19-21 Boulevard Jean Moulin, CEDEX 05, 13385 Marseille, France

**Keywords:** multidrug-resistant bacteria, extensively-drug resistant bacteria, alternative strategy, drug repurposing, old drugs, antibiotic combination

## Abstract

Alternative strategies against multidrug-resistant (MDR) bacterial infections are suggested to clinicians, such as drug repurposing, which uses rapidly available and marketed drugs. We gathered a collection of MDR bacteria from our hospital and performed a phenotypic high-throughput screening with a 1280 FDA-approved drug library. We used two Gram positive (*Enterococcus faecium* P5014 and *Staphylococcus aureus* P1943) and six Gram negative (*Acinetobacter baumannii* P1887, *Klebsiella pneumoniae* P9495, *Pseudomonas aeruginosa* P6540, *Burkholderia multivorans* P6539, *Pandoraea nosoerga* P8103, and *Escherichia coli* DSM105182 as the reference and control strain). The selected MDR strain panel carried resistance genes or displayed phenotypic resistance to last-line therapies such as carbapenems, vancomycin, or colistin. A total of 107 compounds from nine therapeutic classes inhibited >90% of the growth of the selected Gram negative and Gram positive bacteria at a drug concentration set at 10 µmol/L, and 7.5% were anticancer drugs. The common hit was the antiseptic chlorhexidine. The activity of niclosamide, carmofur, and auranofin was found against the selected methicillin-resistant *S. aureus*. Zidovudine was effective against colistin-resistant *E. coli* and carbapenem-resistant *K. pneumoniae*. Trifluridine, an antiviral, was effective against *E. faecium*. Deferoxamine mesylate inhibited the growth of XDR *P. nosoerga*. Drug repurposing by an in vitro screening of a drug library is a promising approach to identify effective drugs for specific bacteria.

## 1. Introduction

The emergence and spread of multidrug-resistant (MDR) pathogens represent a global healthcare question nowadays. Treating infections caused by MDR bacteria can be challenging for clinicians [1,2].

Multi-drug resistance is conventionally defined as resistance to one or more than one agents of three or more antibiotic classes [3]. Pathogens can also be qualified as extensively drug-resistant (XDR) if they remain susceptible to only one or two antimicrobial categories, or pandrug-resistant (PDR) if they are resistant to all of antimicrobial categories routinely tested in the laboratory [3]. The definition of “difficult-to-treat” resistant (DTR) bacteria seems to be more suitable for clinical practice, qualifying Gram negative bacteria when they are resistant to all first-line antibiotics, such as β-lactams or fluoroquinolones [4]. In any case, treatment of MDR bacterial infections can be limited due to resistance to first-line antibiotics and, now, to last-line therapies [5]. Resistant Gram positive cocci has shown resistance to glycopeptides [6]. Carbapenems or polymyxins may become ineffective against Gram negative strains due to the emergence of carbapenemase enzymes or structural changes [6]. Therapeutic impasses due to resistant bacteria are still rare, for now, but in some situations, clinicians must now tackle resistance to treat fragile patients [5]. Strategies to prevent therapeutic impasses are essential, especially for non-fermenting Gram negative bacteria. Indeed, they display a broad intrinsic resistance and are often infectious agents in immunocompromised patients [7]. As a result, many alternative strategies are now considered to optimize the treatment of infectious diseases by MDR bacteria, such as phage therapy, antibodies, probiotics, antivirulence factors, and drug repurposing [8,9].

The objective of drug repurposing is to identify a novel clinical use for an existing drugs approved in clinical medicine for a different indication. This method should be considered for treating new pathogens or agents for which no effective treatment is available [10]. Indeed, current pharmaceutical companies have no economic interest in developing new molecules to treat MDR bacteria that remain isolated cases [10]. As the situation is a public health issue [11], finding effective drugs already in our armamentarium can be an excellent alternative. Moreover, this offers advantages in terms of economic drug development or as an accelerator of the process by skipping preclinical trials [12]. To treat MDR infections, repurposing a drug seems to be an easy and rapid strategy. One of the methods to do this is a phenotypic-based assay, which consists of testing the efficacy of a large panel of drugs on the survival of a bacterium in vitro, as a high-throughput screening [10]. Using this strategy, some efficient drugs have been found so far, such as ribavirin against *Candida* strains [13], the antiretroviral zidovudine against Enterobacteriaceae [14] and the antihelminthic niclosamide against *Staphylococcus aureus* [15]. This strategy allows us to test a drug library at a single concentration that is sometimes higher than the concentration tested in routine testing [16].

Currently, scientific research is mainly directed towards finding new drugs against bacteria that belong to the ESKAPE group (including *Enterococcus faecium*, *S. aureus*, *Klebsiella pneumoniae*, *Acinetobacter baumannii*, *Pseudomonas aeruginosa*, and *Enterobacter* spp.). However, some “intrinsically” multi-drug resistant bacteria remain major challenges to be treated successfully. This is especially the case in cystic fibrosis (CF) patients, particularly after pulmonary transplant when post-transplantation immunosuppressive therapy increases the risk for opportunistic infections [17]. The impact of PDR bacterial pathogens contributes to greater mortality in the immunocompromised CF population because of treatment difficulties [18,19].

The objective of our study was to decipher which drugs are still effective against bacteria that have a high level of drug resistance and pose potential treatment problems. We focused on various MDR or XDR bacteria, especially those found in CF patients. We investigated the action on the bacterial growth of drugs with an untested antimicrobial potential in vitro, which could be a first step towards a new drug repurposing strategy. We gathered a collection of various drug-resistant bacteria from our hospital and performed a phenotypic high-throughput screening with a 1280 FDA-approved drug library.

## 2. Results

### 2.1. Antibiotic Resistance Profile and Genomic Support of Selected Strains Panel

The two MDR Gram positive strains were a clinical strain of methicillin- and glycopeptides-resistant *S. aureus* P1943 carrying the *mecA* gene [20], and a clinical strain of vancomycin-resistance *E. faecium* P5015 carrying a *vanA* gene (Table 1). Among the six Gram negative bacteria tested, three isolates (the *Escherichia coli* DSM 105182 strain [21], *K. pneumoniae* P9495, and *A. baumannii* P1887 [22]) were classified as MDR and three isolates were classified as XDR (*P. aeruginosa* P6540, *Burkholderia multivorans* P6539, and *Pandoraea nosoerga* P8103), as shown in Table 1. Four isolates were carbapenem-resistant (P9495, P1887, P6539, and P8103), including three producing NDM-1 for *A. baumannii*, OXA-48 for *K. pneumoniae,* and OXA-158 for *P. nosoerga*. Five of them were resistant to colistin, a last-line therapeutic drug (all except *A. baumannii*). The *E. coli* DSM 105182 strain carried a plasmid-mediated colistin resistance gene, *mcr-1* (Table 1).

### 2.2. General High-Throughput Screening Results

**Figure 1:**
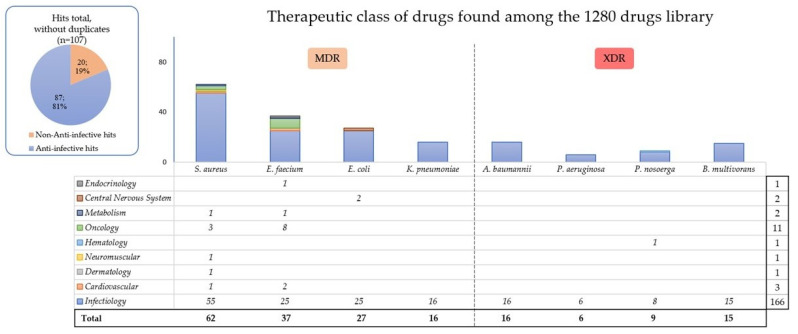
Hits found after an in vitro screening of the 1280 drugs library for each bacterium according to their therapeutic class. On the left, we discarded the duplicates and found 107 hits for all the Gram positive and negative bacteria. Some drugs are classified in various therapeutic classes and can be either antibacterial, antifungal, or antiviral, so we first considered the antibacterial effect in our counting or the major known effect. This chart highlights that most molecules that are active in vitro against bacterial growth are anti-infectives. Only a few options among the 1280 molecules tested can inhibit the bacterial growth of *P. nosoerga* or *P. aeruginosa*. All the compounds per bacterium are detailed in Supplementary Table S1. MDR: multidrug-resistant; XDR: extensively drug-resistant.

Various hits with a growth inhibition rate of 90% or more were found, as shown in Figure 1, and the non-anti-infective compounds are listed for each bacterium in Table 2.

Overall, 62 drugs inhibited at least 90% of the *S. aureus* growth, 37 for *E. faecium*, 27 for *E. coli,* 16 for *K. pneumoniae,* and 16 for *A. baumannii* (Figure 1). Among the XDR bacteria, 6 hits were found for *P. aeruginosa*, 9 for *P. nosoerga,* and 15 for *B. multivorans*.

**Figure 2:**
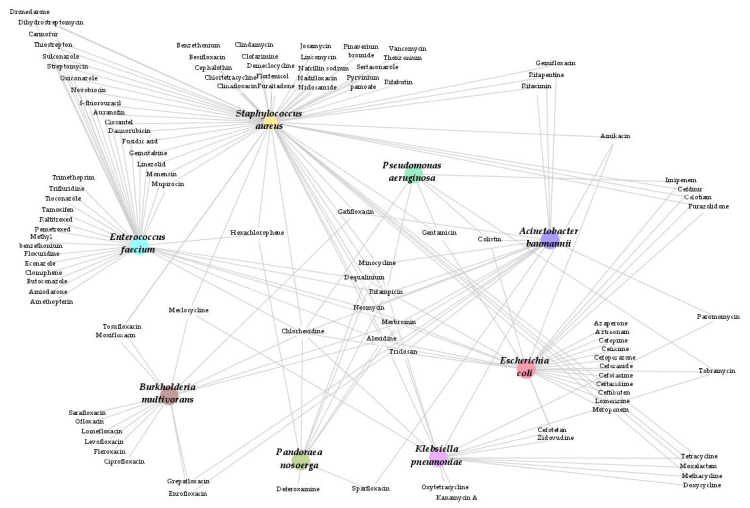
Compounds with a common efficacy on the growth of Gram positive or Gram negative bacteria in this 1280 drugs screening at 10 µmol/L. These molecules must be further tested more carefully, particularly regarding CMI assays, pharmacokinetics and pharmacodynamics, and administration modes in relation to the type of infection, etc.

Overall, among these nine therapeutic classes involved, we found 107 different hits for all the selected Gram positive and Gram negative pathogens in total, including 20 (19%) beyond antimicrobial agents (Figure 1). Among them, eight (7.5%) oncology drugs were found active on Gram positive bacteria, namely: tamoxifen citrate, gemcitabine, carmofur, floxuridine, pemetrexed disodium, raltitrexed, 5-fluorouracil, and methotrexate (Table 2). For the two MDR Gram positive bacteria tested, 23 common hits were found, including an antiarrhythmic (dronedarone hydrochloride), two antiseptics (chlorhexidine and hexachlorophene), twelve antibacterials, an antiprotozoal (monensin sodium salt), an antihelminthic (closantel), an antirheumatic (auranofin), two antifungals (oxiconazole and sulconazole nitrate), and three antineoplastics (5-fluorouracil, carmofur, and gemcitabine; Figure 2). Gram negative bacterial hits all belonged to antimicrobial agents, except two (azaperone and lomerizine hydrochloride) for *E. coli* and deferoxamine mesylate for *P. nosoerga* (Figure 1 and Table 2). Between Gram negative and Gram positive bacteria, the antiseptic chlorhexidine was the only common compound among all of the therapeutic class (Figure 2).

### 2.3. Main Hits by Species

All hits are reported in Figure 2, but we mentioned below those that required our attention for each species.

Various antibiotics (furazolidone and monensin) and antiseptics were effective, but alsoantihelminthics, such as closantel, niclosamide, or pyrvinium pamoate, or antifungals, such as sulconazole, inhibited this *S. aureus*. In addition, the antiarrhythmic dronedarone was also effective against this strain (Table 2). Among the compounds, we noticed that antifungal azoles were effective against *E. faecium* (butoconazole, econazole, oxiconazole, tioconazole, and sulconazole). All these drugs are intended for topical use. Amiodarone, methotrexate, and an antiviral drug, trifluridine, were also found to be effective (Table 2). The same clomiphene citrate and tamoxifen, selective oestrogen receptor modulators, inhibited the growth of the resistant *E. faecium. E. coli* bacterial growth was inhibited by anti-infective drugs such as known antibiotics or antiseptics, but also by an antiviral, zidovudine. The veterinary neuroleptic azaperone and the migraine treatement lomerizine hydrochloride (central nervous system (CNS)) were two non-anti-infective drugs effective against *E. coli* (Table 2).

Antibiotics such as tetracyclines or aminoglycosides and the antiviral zidovudine inhibited the growth of *K. pneumoniae*.

Drugs not belonging to the anti-infectious class showed very limited efficacy in inhibiting the growth of *A. baumannii*. As anti-infectious drugs, fluroquinolones, rifamycin, and antiseptics (merbromin, triclosan, and chlorhexidine) were found to be the most effective against the selected carbapenem-resistant *A. baummanii.* Colistin was also effective at 11.5 mg/L (10 µmol/L).

Against these XDR non-fermenting Gram negative bacteria (*P. aeruginosa, B. multivorans,* and *P. nosoerga*), fewer drugs showed action on their growth, and they were mostly antiseptics or antibiotics. However, this screening may point to potential combination or higher dosage regimens on drugs that are still effective against XDR strains (Table 3). For instance, higher concentrations of colistin were effective against *P. aeruginosa* when it was initially resistant in our routine tests (Table 2). In terms of drug repurposing, by reducing the detection limit to 55%, we were able to find that an anti-rheumatic agent, auranofin, was effective against *P. aeruginosa*. Deferoxamine mesylate was the only non-anti-infective hit against *P. nosoerga* (Table 2).

### 2.4. Comparison with Data from the Literature

We compared our results with pharmacokinetic data from literature studies for each bacterium in order to repurpose our hits (Supplementary Table S2).

The concentration tested for the antiarrhythmic dronedarone in the screening was effective against *S. aureus,* but was much higher than the plasma concentrations found in humans (tested at 5.9 mg/L and found from 0.084 to 0.167 mg/L in human plasma) [23]. However, amiodarone was active as an achievable concentration on *E. faecium* (tested at 6.5 mg/L and found at 1170 mg/L in human plasma). This antibacterial effect on Gram positive bacteria has not been previously demonstrated to the best of our knowledge. The narrow therapeutic window of antiarrhythmics and their side-effects leads to a challenging clinical use and can thus be controversial [24].

Anticancer drugs have also shown some effectiveness against Gram positive bacteria. In this screening, gemcitabine was effective at 2.6 mg/L for *S. aureus* and *E. faecium*. In the literature, the maximum serum concentration (Cmax) could be 2 mg/L with infusion at 600 mg/m^2^ [25] or 40.9 mg/L with an administration of 2000 mg/m^2^ [26].

Previous pharmacokinetics data on deferoxamine mesylate revealed a steady-state concentration of 3.9 mg/L using continuous intravenous deferoxamine infusion at 50 mg/kg/d [27], which is in the same range as our screening concentration (5.6 mg/L–10 µmol/L).

## 3. Discussion

### 3.1. What Are the Potential Therapeutic Options for Treating These MDR Bacteria?

After analyzing the results of this screening of 1280 molecules on a panel of XDR or MDR strains, we found several possible options that could help to fight these bacteria after assessing their effective antibacterial potential and performing additional assays.

### 3.2. To Find a Common Hit

**Figure 3:**
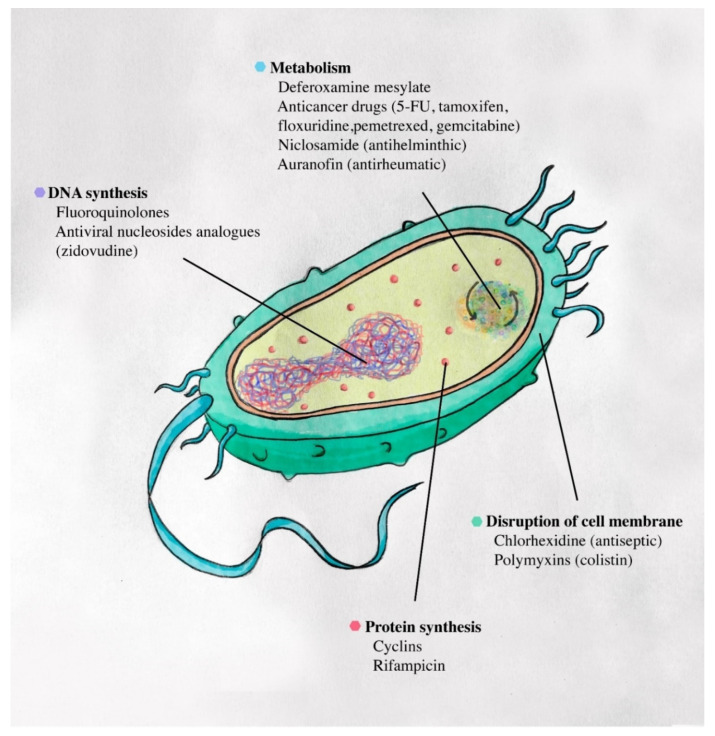
Mechanisms of action of in vitro effective drugs on Gram negative bacteria tested at 10 µM.

The only common hit we found among 1280 drugs for this panel was chlorhexidine. By disrupting the cell membrane and interfering with osmosis [28] (Figure 3), this widely antiseptic and disinfectant is used, for instance, as a topical agent for skin decolonization or to sterilize surgical tools [29]. After all, this common result is in favor of the chlorhexidine bathing recommended before performing a transplant to limit Gram negative bacterial infections [30,31]. Its efficacy among MDR and XDR bacteria is also supported by previous studies on VRE [32], MRSA [33], carbapenemase-producing *K. pneumoniae* [34], or MDR Gram negative [35]. Despite its wide use in prevention, its effectiveness must be monitored for the development of resistance or adverse effects [36]. Furthermore, not finding a common hit for these bacteria for systemic use is not worrisome, as this would also affect the vital microbiota.

### 3.3. To Repurpose a Molecule

In this study, some hits were specifically efficient against bacteria and were not initially marketed for that use: they could be tied to drug repurposing [10]. In this way, the antihelminthic niclosamide, with more than 90% inhibition of *S. aureus*, has already been widely proposed to treat drug-resistant bacteria [15,37,38,39] (Figure 3). Its antibacterial potential has been described for hospital acquired infections as MRSA [15] or intestinal decolonization of VRE [39], but also in combination with colistin to treat colistin-resistant Gram negative bacilli infections [37,38]. As carmofur and auranofin are also effective against MRSA, they have been further investigated for their anti-biofilm activity [40]. The efficacy of clomiphene on *E. faecium* confirmed the idea of repurposing some oestrogen receptor antagonists to treat infectious diseases [41]. In addition, the antimetabolite methotrexate was effective against the growth of *E. faecium* and could act by inhibiting dihydrofolate reductase, such as trimethoprim, which was also effective. An old study conducted in 1974 reported methotrexate activity with a MIC of 0.15 µg/L against *Streptococcus faecium* [42], formerly named *E. faecium*, confirmed on *S. faecalis* ten years after [43]. The antibacterial effect of zidovudine on *E. coli* and *K. pneumoniae*, previously shown in 1987 [44], confirmed our report about its potential use to treat infections on colistin-resistant Gram negative bacteria [14].

However, drug repurposing is not feasible for all cases. Concerning *E. coli*, the veterinary use of azaperone [45] and the low serum concentration for lomerizine in humans [46] do not allow these CNS drugs for immediate clinical use, but require further investigations (Supplementary Table S2). Paromomycin, an aminoglycoside effective against *K. pneumoniae,* is usually used to treat parasite infections such as amebiasis. Nevertheless, like neomycin, these aminoglycosides display a poor intestinal absorption, which is not suitable for systemic infections. Paromomycin can still be used for the intestinal decolonisation of resistant *Enterobacteriaceae* [47]. Furthermore, colistin and methotrexate are compounds with known and feared adverse effects in clinical practice.

### 3.4. Using Drug Associations

A combination of drugs consisting of old drugs, broad spectrum antibiotics, and non-anti-infective compounds, may provide killing effects [48]. Rifampicin and minocycline, effective on *P. nosoerga*, should be verify the effectiveness of the combination with a checkerboard assay, as it already showed goods results [49]. Fluoroquinolones in combination with β-lactams are also promising, as shown by in vitro testing against *Burkholderia cepacian* [50] or evaluation in a large cohort study against Gram negative bacilli bacteraemia [51].

Combination therapy is recommended for MDR bacteria treatment [10]. It allows clinicians to use drugs at lower concentrations than in monotherapy through a synergistic effect, as shown by Lee J. H. et al. with rifampicin and colistin against MDR *A. baumannii* [52]. In vitro studies reported a good efficiency of imipenem in combination with rifampicin or colistin against MDR clinical isolates of *P. aeruginosa* [53,54]. Furthermore, auranofin, which was effective against the selected XDR *P. aeruginosa*, has been studied in combination with polymyxins on Gram negative species that permeabilize the outer membrane of Gram negative and help auranofin enter the bacteria cell (Figure 3). The combination with colistin on *P. aeruginosa* showed a synergistic effect [55] and the addition of ceftazidime inhibited > 80% growth of 10 MDR pathogens, including *P. aeruginosa* [56].

Trifluridine, an antiviral molecule, is used in monotherapy as anti-herpesvirus eye drops. It has a low bioavailability after clinical administration as it is rapidly degraded via thymidine phosphorylase. Therefore, in combination with a potent thymidine phosphorylase inhibitor, tipiracil has been studied for antineoplastic use [57]. This combination could be tested on resistant *Enterococcus* sp. against systemic infections.

Deferoxamine mesylate is used to treat iron overdose as hemochromatosis because it binds iron and aluminium. This drug is effective against *P. nosoerga* and is not known to date as being used for antibacterial monotherapy. By complexing with aluminum or gallium, deferoxamine has a Trojan horse effect, carrying these toxic metal ions into the bacterial metabolism [58] (Figure 3). Moreover, when used with ascorbic acid, deferoxamine showed a bacterial growth inhibiting effect [59], and added to gentamicin, this triple combination displayed a synergistic effect against some *E. coli* strains [60]. As it is easy to apply, this association should be assessed by performing MIC assays on these strains.

### 3.5. Damaging Basic Mechanisms of Bacteria

Although using old drugs or combinations of drugs can be successful, drugs acting on essential mechanisms such as DNA or the protein cycle of all bacteria are frequently represented (Figure 3).

In addition, our screening concentration was of four oncology molecules in the human concentration range (Supplementary Table S2). Some activities have been already reported, like 5-fluorouracil, tamoxifen, floxuridine, pemetrexed disodium, and gemcitabine in Gram positive or in resistant Gram negative strains [10,61,62]. A study testing tamoxifen in combination with polymyxins against XDR Gram negative pathogens used an achievable concentration for oral administration [62]. However, we are aware that anti-cancer drugs would not only target bacteria, but would also regulate immunity and host response [63]. As these drugs can disrupt the immune response, it is doubtful whether their use to fight infectious diseases would be beneficial. In a review by Soo V. et al., similarities between cancer cells and bacterial infections encouraged the use of agents with a broad activity, which act on the cell cycle [64] (Figure 3). Society might be frightened to try such therapies due to the many side effects of treating infectious diseases. However, given our results, using anticancer drugs might be the only solution to treat patients infected with a XDR bacterium for which no treatment is available, despite their toxicity [16].

On the other hand, fluoroquinolones and tetracyclines were effective in most of our panel. These antibiotics have a broad spectrum, with activity on Gram positive and Gram negative bacteria. Cyclins inhibit protein synthesis and fluoroquinolones prevent DNA replication and transcription—these mechanisms are essential for bacteria to live and are therefore highly conserved in all species (Figure 3). However, bacteria can adapt quickly to these antibiotics, developing several resistance mechanisms, leading to cross-resistance and increasing MIC [65]. The selected *B. multivorans* was previously ciprofloxacin-resistant with disk diffusion, but became susceptible in the screening test, where the concentration tested was 3.31 mg/L (10 µmol/L). This difference was due to the concentration used in the screening method being higher than the usual dose used in sensitivity testing and medical practice. Using a higher concentration of ciprofloxacin or other fluoroquinolones could treat *B. multivorans* infection (Table 3). This is in accordance with previous successful studies with an increase of dosage regiments against PDR bacteria [66]. After assessment of the risks and benefits of the treatment, this could lead to a better clinical response and circumvent resistance [16].

Finally, few other anti-infective agents were effective pointing essential mechanisms. Antiviral nucleosides analogues like trifluridine (*E. faecium*) or zidovudine (against *Enterobacteriaceae* [14]) targeted the inhibition of DNA synthesis, which could explain their effect on bacterial inhibition growth (Figure 3). In addition to having some synergy with antibiotics, nucleoside analogues should be considered as new weapons against bacterial infection, as suggested by A. E. J. Yssel et al. [61,67].

### 3.6. Personalizing the Treatment in a Compassionnal Approach

Access to a rapid screening method for many commercially available molecules is a method that can help clinicians propose a therapeutic option to patients when faced with a therapeutic impasse. This method, which should be limited to the most severe cases, allows for the identification of molecules of interest, for which the MIC must be checked to assess whether it is achievable in vivo. Similarly, the in vivo efficacy of the identified molecules will depend on the data in the literature. For instance, this could be a solution for opportunistic bacteria that have many resistance determinants such as *Burkholderiales* [68]. The clinical impact of this screening can be significant, as these molecules have already been approved and marketed and they can be quickly made available to clinicians. The risk−benefit of patients facing such infections must be assessed as a compassionnal treatment, as well as the management of certain adverse effects and the toxicity that can be controlled [16]. This strategy has been applied to the emergence of new pathogens for which no treatment is yet available because of the SARS-CoV-2 outbreak [69].

In this study, we investigate the in vitro efficacy of 1280 FDA-approved drugs among eight MDR and XDR clinical bacteria displaying various resistance phenotypes and resistance genes. We screened a library of 1280 drugs against a collection of bacteria. A total of 107 compounds from nine different therapeutic classes inhibited > 90% growth of the chosen Gram negative and Gram positive bacteria. In view of the hits found, we noticed that the difficulty in finding active compounds on Gram negative bacteria is greater than for Gram positive bacteria. This reflects our clinical practice issues with the MDR Gram negative bacteria, and especially the non-fermenting Gram negative.

Some of these results represent a first step towards the use of a drug repurposing strategy for the treatment of MDR bacterial infections. Nevertheless, these molecules must be further studied on a case-by-case basis to verify their antibacterial efficacy, understand their mechanisms of action, and confirm their suitability for a relevant administration. Assessing a potential antibacterial must use reference bacterial isolates for obtaining the MIC range, MIC50, and MIC90. This protocol is, after all, becoming established in our laboratory as clinical screening for special patients with XDR bacterial infection. Finally, it might be judicious to create a network of different fields of expertise to promote the investigation of these new options, so that each option can be explored in detail, with all the necessary analyses to achieve the final goal—finding new therapeutic solutions to deal with antibiotic resistance.

## 4. Materials and Methods

### 4.1. Collection and Susceptibility of Bacterial Strains

With the exception of the *mcr-1* reference strain *E. coli* (DSM 105182), all strains (*E. faecium* P5014, *S. aureus* P1943, *A. baumannii* P1887, *K. pneumoniae* P9495, *P. aeruginosa* P6540, *B. multivorans* P6539, and *P. nosoerga* P8103) were collected for their antibiotic resistance profile from clinical samples. To determine antibiotic susceptibility, the standard disk diffusion method and breakpoint assessment were performed according to the European Committee of Antimicrobial Susceptibility Testing (EUCAST) recommendations. When breakpoints were not available in EUCAST, we used recommendations from the French Society of Microbiology (CA-SFM). We used the previously published definitions from the study of A. Magiorakos [3] to categorize the MDR (acquired non-susceptibility to at least one agent in three or more antimicrobial categories) and XDR (non-susceptibility to at least one agent in all but two or fewer antimicrobial categories) phenotypes.

### 4.2. Preparation of Isolates

The isolates were inoculated on a TSA medium (Trypticase soy agar, BioMérieux, Marcy-l’Étoile, France) at 37 °C for 18–24 h. The strains were suspended at 10^8^ CFU/mL in 0.9% NaCl and diluted 1/100 (10^6^ CFU/mL) in cation adjusted Mueller–Hinton broth media (CAMHB, Merck KGaA, Darmstadt, Germany).

### 4.3. High-Throughput Screening Assay

The screening test was performed on 96-well microplates with 1280 FDA-approved drugs from a chemical library (Prestwick-Chemical, Illkirch Graffenstaden, France), in which 16% of the drugs were initially used for the nervous system, 14% for the cardiovascular system, and 14% of the anti-infectives for systemic use. Regardless of the protocol, the final drug concentration in each well was always 10 µmol/L. Dilution of the initial plates was performed to achieve a DMSO (dimethyl sulfoxide) concentration of less than 0.1% in all of the final plates (initial concentration of 10%).

The plates were prepared in duplicates with 80 µL of CAMHB, 10 µL of drugs at 100 µmol/L, and 10 µL of each bacterium at 10^6^ CFU/mL, and with negative and positive controls, incubated at 37 °C for 18–24 h shaking at 300 rpm.

Bacterial growth was measured by spectrophotometry (Multiskan Spectrum, Thermo Fisher Scientific, Waltham, MA, USA). Then, the bacterial growth inhibition rate was calculated with the following equation as a function of absorbance (*A*) in the wells:

$$Bacterial\;growth\;inhibition\;\left(\%\right)=\left[1-\left(\frac{A_{sample}-A_{negative\;control}}{A_{positive\;control}-A_{negative\;control}}\right)\right]\times 100$$

Hits were selected when the growth inhibition rate was greater than 90%.

## Figures and Tables

**Table 1:** Antimicrobial susceptibility profiles and characterization from the selected panel according to the study of Magiorakos et al. [3]. MDR: multidrug-resistant; XDR: extensively drug-resistant.

Strain and No.	Characterization [3]	Non-Susceptibility to at Least One Agent in All Those Classes	Known Resistance Genes
*S. aureus* P1943 [20]	MDR	Fluoroquinolones, anti-staphylococcal β-lactams, glycopeptides, and macrolides	*mecA, gyrA, aaD, bleO,* and *ermC*
*E. faecium* P5015	MDR	Aminoglycosides, glycopeptides, and tetracyclines	*vanA*
*E. coli* DSM 105182 [21]	MDR	Polymyxins, tetracyclins, and fluoroquinolones	*mcr-1*
*K. pneumoniae* P9495	MDR	Aminoglycosides, penicillins + β-lactamase inhibitors, carbapenems, extended-spectrum cephalosporins, fluoroquinolones, folate pathway inhibitors, monobactacms, and polymyxins	*bla*_OXA-48_
*A. baumannii* P1887 [22]	MDR	Aminoglycosides, carbapenems, fluoroquinolones, penicillins + β-lactamase inhibitors, extended-spectrum cephalosporins, and folate pathway inhibitors	*bla*_OXA-51_*, bla*_OXA-23_*,* and *bla*_NDM-1_
*P. aeruginosa* P6540	XDR	Aminoglycosides, antipseudomonal cephalosporins, antipseudomonal fluoroquinolones, penicillins + β-lactamase inhibitors, monobactacms, and polymyxins	
*B. multivorans* P6539	XDR	Aminoglycosides, carbapenems, cephalosporins, fluoroquinolones, penicillins + β-lactamase inhibitors, monobactacms, folate pathways inhibitors, glycylcyclines, and polymyxins	
*P. nosoerga* P8103	XDR	Aminoglycosides, carbapenems, cephalosporins, fluoroquinolones, penicillins + β-lactamase inhibitors, rifamycins, folate pathways inhibitors, tetracyclins, phosphonic acids, and polymyxins	*bla*_OXA-158_

**Table 2:** Hits that are not in the “infectiology” therapeutic class but in all other classes.

Name of Strain	Hits Except “Infectiology” Class
*S. aureus* P1943	Dronedarone hydrochloride (cardiovascular); thonzonium bromide (dermatology); auranofin (metabolism); pinaverium bromide (neuromuscular); and 5-fluorouracil, carmofur, and gemcitabine (oncology)
*E. faecium* P5015	Amiodarone and dronedarone hydrochloride (cardiovascular); clomiphene citrate (Z and E) (endocrinology); auranofin (metabolism); tamoxifen citrate, gemcitabine, carmofur, floxuridine, pemetrexed disodium, raltitrexed, 5-fluorouracil, and methotrexate (oncology)
*E. coli* DSM 105182	Azaperone and lomerizine hydrochloride (central nervous system)
*P. nosoerga* P8103	Deferoxamine mesylate (hematology)

**Table 3:** Possible alternative solutions for the selected XDR strains with in vitro activity. If the doses of these antibiotics are increased, it is to consider improved efficacy. However, this must be monitored if the dosages would be beyond the recommendations (for renal function, serum concentrations, etc.) and these molecules are not recommended for use as monotherapy.

	Combination Therapy	Drug Repurposing	Increased Dosages
*P. aeruginosa*	Rifampicin + imipenem Colistin + imipenem	Auranofin (+ colistin ± ceftazidime)	Colistin
*P. nosoerga*	Rifampicin + minocyclin	Deferoxamine + ascorbic acid (+ gentamicin)	Rifampicin
*B. multivorans*	Fluoroquinolones + β-lactams	-	Fluoroquinolones

## Supplementary Materials

The following supporting information can be downloaded at: https://www.mdpi.com/article/10.3390/antibiotics11030291/s1, Table S1: Molecules and their therapeutic class with a bacterial growth inhibition percentage higher than 90% for the bacteria tested, Table S2: Comparison of non-anti-infective hits with human PK data from literature. (References [70,71,72,73,74,75,76,77,78] are cited in the supplementary materials).
