# Supplementary material for: In Vitro Screening of a 1280 FDA-Approved Drugs Library against Multidrug-Resistant and Extensively Drug-Resistant Bacteria
Source: Antibiotics (Basel). 2022 Feb 22;11(3):291. doi: 10.3390/antibiotics11030291 (PMC8944690; doi:10.3390/antibiotics11030291)
Supplement: Supplementary file 1 [file antibiotics-11-00291-s001.zip › Table S2 Annex 2 - data PK proof LP.pdf]

| | Therapeutic class | Hits except "infectiology" class | PM g/mol | Tested C° in mg/l (=10 µmol/l) | | *Data from in vivo studies* | References |
|---|---|---|---|---|---|---|---|
| *S. aureus* P1943 | Cardiovascular | Dronedarone hydrochloride | 593.2 | 5.9 | | Steady-state plasma concentrations of **84 to 167 ng/mL (84/167 µg/l)**. with 400 mg twice daily | [22] |
| | Dermatology | Thonzonium bromide | 591.7 | 5.9 | | *external use in an antibiotic suspension. any data about serum concentration* | |
| | Metabolism | Auranofin | 679.5 | 6.8 | | Cmax: **0.025 µg/ml (0.025 mg/l)**. after 6mg per day | [39] |
| | Neuromuscular | Pinaverium bromide | 591.4 | 5.9 | | Cmax: **3.609.98 pg/mL (3.6 µg/l)** for 100 mg. with a bioavailability very low | [70] |
| | Oncology | 5-fluorouracil | 130.0 | 1.3 | | Cmax: between **393.8 - 487 ng/ml (440.4 µg/l)** for a dose of 50 mg/kg | [71] |
| | | Carmofur | 257.3 | 2.6 | | Serum concentration: lower than **0.02 µg/ml (0.02 mg/l)**. with daily administrations of 600 mg for more than 28 days | [72] |
| | | Gemcitabine | 263.2 | 2.6 | Go further | Cmax: **40.9 µg/ml (40.9 mg/l)** with administration of 2000mg/m² or **7.71 µmol/ml (2 mg/l)** with infusion at 600 mg/m² | [24,25] |
| *E. faecium* P5015 | Cardiovascular | Amiodarone | 645.3 | 6.5 | Go further | Cmax: **1.17 mg/ml (1.17 g/l)**. after oral dose of 600 mg | [73] |
| | | Dronedarone hydrochloride | 593.2 | 5.9 | | Steady-state plasma concentrations of **84 to 167 ng/mL (84/167 µg/l)**. with 400 mg twice daily | [22] |
| | Endocrinology | Clomiphene citrate (Z.E) | 405.96 | 4.1 | | Cmax: **15 ng/mL (15 µg/l)** after single oral dose of 50 mg | [74] |
| | Metabolism | Auranofin | 680.5 | 6.8 | | Cmax: **0.025 µg/ml (0.025 mg/l)**. after 6mg per day | [39] |
| | Oncology | Tamoxifen citrate | 371.5 | 3.7 | | Cmax: **368 nmol/l (136.7 µg/l)** for a dose of 20 mg | [75] |
| | | Gemcitabine | 263.2 | 2.6 | Go further | Cmax: **40.9 µg/ml (40.9 mg/l)** with administration of 2000mg/m² or **7.71 µmol/ml (2 mg/l)** with infusion at 600 mg/m² | [24,25] |
| | | Carmofur | 257.3 | 2.6 | | Serum concentration: lower than **0.02 µg/ml (0.02 mg/l)**. with daily administrations of 600 mg for more than 28 days | [72] |
| | | Floxuridine | 249.2 | 2.5 | | Cmax: **4.90 ng/ml (4.90 µg/l)**. 400 mg three times daily. If haemodialysis. less. | [24] |
| | | Pemetrexed disodium | 470.4 | 4.7 | Go further | Cmax: **119 µg/mL (119 mg/l)** for 500 mg/m² by infusion | [76] |

| | | | | | | | |
|---|---|---|---|---|---|---|---|
| | | Raltitrexed | 458.0 | 4.6 | | Cmax: **652.9 ng/ml (0.6 mg/l)** with single intravenous dose of 3 mg/m² | [77] |
| | | 5-fluorouracil | 130.0 | 1.3 | | Cmax: between **393.8 - 487 ng/ml (440.4 µg/l)** for a dose of 50 mg/kg | [71] |
| | | Amethopterin (Methotrexate) | 454.4 | 4.5 | | Cmax: **0.01 - 0.1 µmol/l (4.5 - 45.4 µg/l)** with oral doses of 10-15 µg | [78] |
| *E. coli* DSM 105182 | Central Nervous System | Azaperone | 327.4 | 3.3 | | *veterinary drug. no PK data on humans* | |
| | | Lomerizine hydrochloride | 468.5 | 4.7 | | Cmax: **9.06 µg/l** after a dose of 10mg | [46] |
| *P. nosoerga* P8103 | Hematology | Deferoxamine mesylate | 560.7 | 5.6 | | Cmax: **80 - 130 pmol/L (44.8 - 728.9 ng/l)** with IV bolus of 10 mg/kg | [26] |
